# A dual-task segmentation network based on multi-head hierarchical attention for 3D plant point cloud

**DOI:** 10.3389/fpls.2025.1610443

**Published:** 2025-07-22

**Authors:** Dan Pan, Baijing Liu, Lin Luo, An Zeng, Yuting Zhou, Kaixin Pan, Zhiheng Xian, Yulun Xian, Licheng Liu

**Affiliations:** ^1^ School of Electronics and Information, Guangdong Polytechnic Normal University, Guangzhou, China; ^2^ School of Information Engineering, Guangdong University of Technology, Guangzhou, China; ^3^ School of Computer Science and Technology, Guangdong University of Technology, Guangzhou, China; ^4^ Guangzhou Huitong Agricultural Technology Co., Ltd., Guangzhou, China; ^5^ Guangzhou iGrowLite Agricultural Technology Co., Ltd., Guangzhou, China

**Keywords:** automated plant phenotyping, 3D point cloud segmentation, multi-head attention, instance segmentation, semantic segmentation, Multi-Value Conditional Random Field (MV-CRF)

## Abstract

**Introduction:**

The development of automated high-throughput plant phenotyping systems with non-destructive characteristics fundamentally relies on achieving accurate segmentation of botanical structures at both semantic and instance levels. However, most existing approaches rely heavily on empirically determined threshold parameters and rarely integrate semantic and instance segmentation within a unified framework.

**Methods:**

To address these limitations, this study introduces a methodology leveraging 2D image data of real plants, i.e., Caladium bicolor, captured using a custom-designed plant cultivation platform. A high-quality 3D point cloud dataset was generated through reconstruction. Building on this foundation, we propose a streamlined Dual-Task Segmentation Network (DSN) incorporating a multi-head hierarchical attention mechanism to achieve superior segmentation performance. Also, the dual-task framework employs Multi-Value Conditional Random Field (MV-CRF) to enable semantic segmentation of stem-leaf and individual leaf identification through the DSN architecture when processing manually-annotated 3D point cloud data. The network features a dual-branch architecture: one branch predicts the semantic class of each point, while the other embeds points into a high-dimensional vector space for instance clustering. Multi-task joint optimization is facilitated through the MV-CRF model.

**Results and discussion:**

Benchmark evaluations validate the novel framework’s segmentation efficacy, yielding 99.16% macro-averaged precision, 95.73% class-wise recognition rate, and an average Intersection over Union of 93.64%, while comparative analyses confirm its superiority over nine benchmark architectures in 3D point cloud analytics. For instance segmentation, the model achieved leading metrics of 87.94%, 72.36%, and 71.61%, respectively. Furthermore, ablation studies validated the effectiveness of the network’s design and substantiated the rationale behind each architectural choice.

## 1 Introduction

Plant phenotyping captured the interaction between genotype and environment, and encompassed traits essential for crop improvement and for understanding the relationships among genome, environment, and phenotype (Pan, 2015). Despite advances in genotyping, phenotyping tools were often manual, invasive, and time-consuming, which highlighted the need for automated, high-throughput solutions (Song et al., 2025). Computer vision, particularly Deep Learning (DL) methods, advanced phenotyping by integrating feature extraction and decision-making and enabled efficient trait measurement (Magistri et al., 2020; Kolhar and Jagtap, 2021; Li et al., 2023; Murphy et al., 2024).

However, 2D image-based methods often failed in occlusion scenarios and overlooked organ-level segmentation, which was critical for precise phenotypic measurements. For various trait measurements, such as stem length and branch diameter (Turgut et al., 2022), accurately segmenting a plant into its constituent organs was essential. 3D point cloud segmentation, enabled by advancements in 3D photogrammetry and sensing technologies, provided occlusion-free models (Turgut et al., 2022; Li et al., 2022; Akhtar et al., 2024) for precise organ-level analysis, and overcame challenges of 2D methods.

While recent advances in 3D DL-based point cloud segmentation methods showed great potential for enhancing the robustness and precision of point cloud segmentation, their application to full 3D segmentation of plant organs remained limited. The main challenges limiting the wider adoption of 3D deep learning for plant phenotyping included the limited availability of annotated point cloud datasets from real plant models, the need for CNNs specifically designed to handle unstructured and unordered point cloud data, and the complexity of developing networks capable of performing versatile and comprehensive point cloud segmentation. Furthermore, the network struggled to effectively balance organ-level semantic segmentation and instance segmentation.

This study is part of a larger research initiative focused on developing a high-speed, automated platform for plant phenotyping, such as *C. bicolor*. This platform is designed to capture comprehensive plant phenotype data with speed and efficiency.

1. 2D Image Capture: Plants are positioned on a turntable, which rotates at a constant speed. Cameras are mounted at three distinct heights on a stationary support frame, enabling simultaneous image acquisition from multiple perspectives. This configuration ensures complete 360-degree coverage of each plant, eliminating blind spots. As a result, the system captures 180 high-resolution 2D images per plant.
2. 3D Reconstruction: A Structure-from-Motion (SfM) algorithm (Schonberger and Frahm, 2016) processes a sequence of 2D images to generate a 3D point cloud of the plant.
3. Point Cloud Denoising: The point cloud is then denoised using various filters to remove irrelevant points, retaining only those within the target plant region.
4. Semantic and instance Segmentation: The cleaned point cloud is segmented to isolate individual components, such as the stem, leaves, flower pot, and auxiliary markers.
5. Point Cloud Completion (if necessary): To address missing regions due to occlusions or reconstruction artifacts, the segmented point clouds of stems and leaves are completed to achieve a full geometric representation of these structures.
6. Registration: For each plant, point clouds captured at different time points are aligned using non-rigid registration techniques. This process ensures that the plant point clouds from different time points are consistently mapped into the same spatial coordinate system, enabling accurate temporal analysis of plant growth and morphological changes.
7. Phenotype Data Extraction: Phenotypic traits, such as leaf surface area, perimeter, bounding box dimensions, and stem height, are derived from the segmented point clouds. By leveraging the paired temporal information obtained in Step 6, time-series data of the plant’s phenotypic characteristics are produced, enabling systematic investigation of developmental patterns and structural evolution.

This research initiative focuses on the phenotypic characterization and growth prediction of greenhouse plants. The platform enables the collection of plant growth pattern data under varying resource conditions (light intensity, temperature, nutrient content), thereby identifying optimal environmental parameters for crop cultivation. Additionally, it allows us to predict whether current plants require supplementation of growth resources. Going forward, we will utilize drones to capture 3D point clouds of field crops, extending this technology to open-field applications. Naturally, plant occlusion issues in field conditions present greater challenges than those in greenhouse environments. This will be a primary research focus in our future work. Previously, we proposed a multi-scale geometry-aware point-transformer-based plant point cloud completion network to address occlusion issues in tropical ornamental plants (Zeng et al., 2022).

This paper primarily focuses on the fourth step: performing semantic and instance segmentation on the plant point cloud. This step is crucial for enabling steps 6 and 7. First, due to the non-rigid deformations that occur as plants grow, registering point clouds based solely on the plant’s structure is both time-consuming and inefficient. By utilizing segmented registration markers as fixed points and key reference frames, the accuracy and efficiency of non-rigid registration are greatly enhanced. Second, during the process of quantifying plant morphological features, the minimum bounding box technique is commonly applied to analyze key structural attributes. This approach depends on precise instance segmentation to effectively distinguish individual organs, such as stems and leaves. Precise segmentation is therefore critical to ensuring the reliability and accuracy of subsequent phenotypic measurements.

Traditional computer vision-based instance segmentation methods frequently necessitate extensive manual parameter tuning to adapt to different plant species, thereby creating constraints in operational efficiency. Such constraints create barriers to fulfilling the requirements of high-speed, automated plant phenotype measurement. This study presents an innovative point cloud segmentation approach leveraging a Multi-head Hierarchical Attention mechanism, termed the Dual-Task Segmentation Network (DSN). This approach can efficiently detect *C. bicolor*’s stems and individual leaves, laying the foundation for rapid, automated plant phenotype measurement. As for step 5, our preliminary research findings have already been published in (Zeng et al., 2022). Concurrently, we are actively pursuing further research on step 6 to enhance the overall framework.

In this study, we achieved 3D reconstruction from 2D image data of actual plant specimens captured through an automated phenotypic platform, from which we obtained a dataset of 276 instances of annotated *C. bicolor* point clouds. We also make this dataset openly available upon reasonable request to contribute to addressing the limited availability of ornamental plant point cloud datasets.

This research focuses on achieving complete automation and intelligent processing in plant phenotyping. While the current stage of our work has not extensively addressed the issue of leaf occlusion, this challenge will be a key focus in our future research efforts. Our current research not only demonstrates the high-precision performance of the DSN model on this dataset, but also evaluates its advantages in plant organ segmentation through comparisons with leading deep-learning frameworks for point cloud processing, including PointNet, PointNet++ (Qi et al., 2017b; Wang et al., 2019b), DGCNN, and ASIS (Wang et al., 2019a). The key innovations and contributions of this study are summarized as follows:

1. Designing the DSN: We developed a dual-task, point-based deep learning network designed to directly process fully annotated 3D point cloud datasets. DSN simultaneously generates semantic labels and instance embeddings, enabling precise organ-level segmentation. To further refine predictions, we incorporated the Multi-Value Conditional Random Field (MV-CRF) model for joint optimization of object categories and instances, significantly improving segmentation accuracy and phenotypic trait extraction. Our approach achieves state-of-the-art performance in plant phenotyping, with DSN surpassing nine existing deep learning frameworks in both semantic and instance segmentation, demonstrating exceptional accuracy (99.16% overall) and robustness.
2. Proposing Multi-Scale Feature Extraction and Attention Mechanism: Within the DSN, we defined organized local regions based on metric radii to extract multi-scale features, enhancing the flexibility and selectivity of plant geometric modeling. Additionally, we introduced a Multi-head Hierarchical Attention Module (MHAM) to capture feature dependencies between local and global regions. Through ablation studies, we demonstrated the effectiveness of the Local Attention Module (LAM) and Global Attention Module (GAM) within the MHAM of the DSN architecture.
3. Developing and publicly releasing a real plant point cloud dataset for semantic and instance segmentation tasks: We developed a non-destructive, automated phenotypic measurement platform and leveraged 2D image data from real plants for 3D reconstruction, creating a manually annotated point cloud dataset that is publicly available. This dataset includes semantic segmentation labels for three categories (non-plant, leaf, and stem) as well as instance segmentation labels for individual leaves, providing a valuable resource for plant phenotyping research.

## 2 Materials and methods

### 2.1 Overview of the method

In initiating this research, we believed that starting with structurally simple plants would facilitate the gradual application of this technology to more complex plant morphologies, such as soybeans and corn. We selected *C. bicolor* (a member of the Araceae family) as a representative species. Renowned for its vibrant foliage, high ornamental value, and low maintenance requirements, *C. bicolor* is a popular indoor plant. Its short growth cycle and simple structure made it an ideal candidate for this study, allowing for detailed monitoring of its growth through the automated phenotyping platform. Accordingly, we continuously recorded and observed 252 C*. bicolor* specimens over a three-month period, tooking multi-angle pictures of each plant at three-day intervals.

The research was conducted in a greenhouse in Guangzhou, Guangdong Province, China. Using high-precision 3D point cloud data, the DSN model was employed to perform semantic segmentation of *C. bicolor*’s organs (e.g., stems and leaves) alongside instance segmentation specifically for its leaves. In the semantic segmentation task, each point is classified into one of three categories: non-plant (not part of the plant), leaf, or stem. For leaf instance segmentation, our method can accurately identify all points associated with each leaf respectively.

In pursuit of this objective, a system was developed for acquiring 2D images of *C. bicolor*, which were then used for 3D reconstruction to generate a point cloud dataset. We then removed the background and noise points from the point clouds. Additionally, the segmentation tool in CloudCompare (Girardeau-Montaut, 2016) was employed to manually annotate the preprocessed point clouds, creating a 3D point cloud dataset from real plants for neural network training. Subsequently, we scanned the point cloud using overlapping windows and passed it through the DSN to assign semantic classifications. These points were subsequently mapped into a multidimensional embedding space, enabling the clustering of points into distinct object instances. Finally, we propose a Multi-Value Conditional Random Field (MV-CRF) model that holistically embeds the co-optimization of semantic categorizations and instance delineation within a unified framework. This model is constructed through mean field variational inference methodologies.

### 2.2 Data acquisition

#### 2.2.1 Image-capturing system

**Figure 1:**
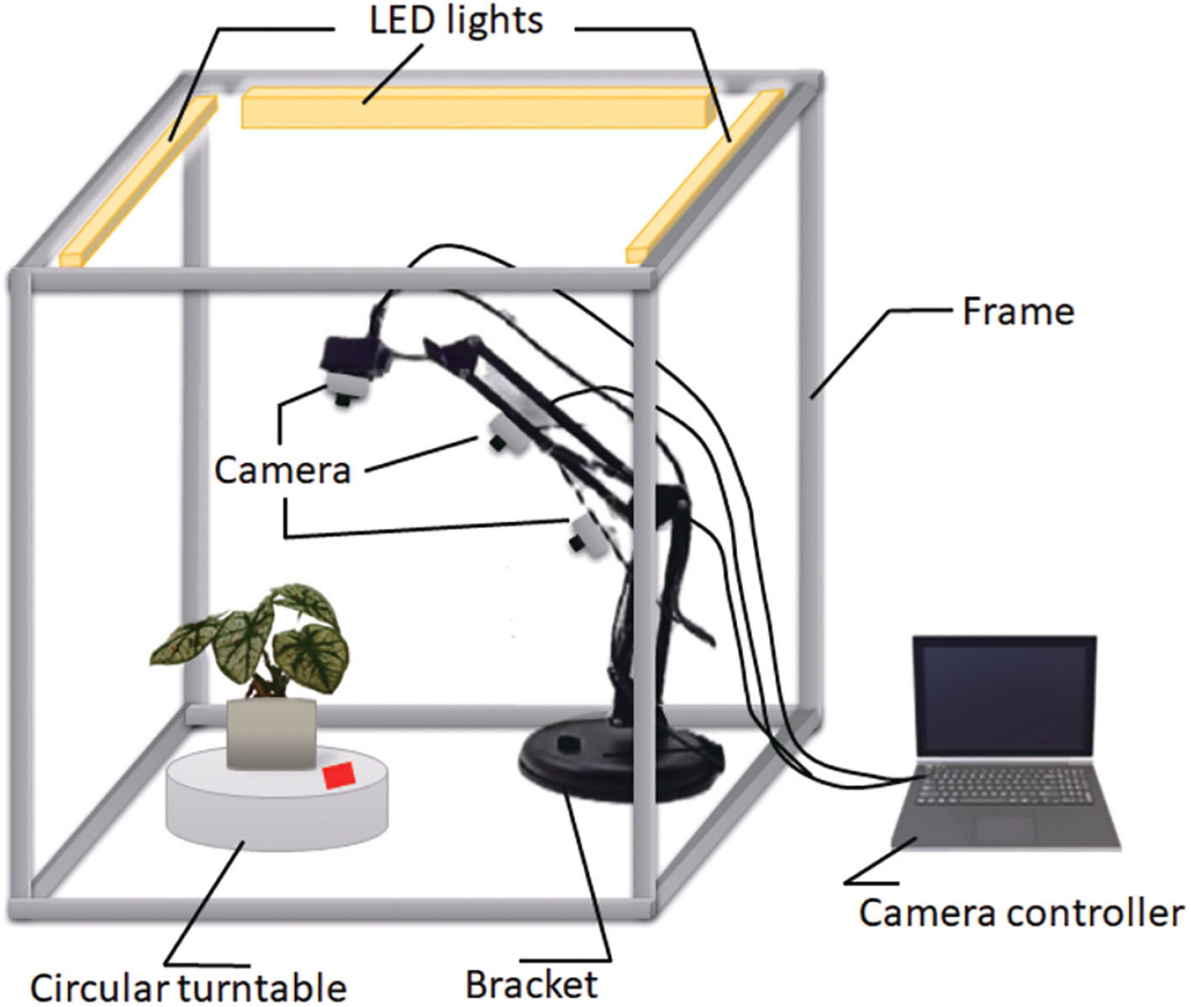
Image capturing system.

The image-capturing system consists of the following key components: a frame, a turntable, a bracket, three LED lights, three digital cameras, a light controller, and a computer that functions as the camera controller, as illustrated in **Figure 1**. The frame (0.8m × 0.8m) was constructed using twelve aluminum extrusions onto which all components were mounted. A circular turntable was engineered to rotate the plant along a predefined trajectory, completing one full revolution every 30 seconds. The bracket securely held the cameras in place, while three LED lights were mounted onto the structural framework to ensure uniform illumination across the imaging area. Three digital cameras were strategically positioned at different angles and set to autofocus throughout the capture process, maintaining platform stability with fixed camera parameters. A Python script was developed to control each camera, capturing 60 images per plant, resulting in a total of 180 images from the tri-camera system. All images were recorded at a resolution of 2048×1536 pixels and saved in JPG format.

#### 2.2.2 3D reconstruction

All 2D images acquired through the imaging apparatus were employed to generate a 3D point cloud through a multi-step 3D reconstruction process. The SfM algorithm was used to reconstruct a dense point cloud through matching, expansion, and filtering processes.

In the first step, the Scale-Invariant Feature Transform algorithm (Lowe, 1999) was employed to extract local feature points. The Euclidean distances between feature points in image pairs were calculated to achieve stereo matching and establish corresponding point pairs. In the second step, the camera’s internal calibration attributes and spatial orientation (pose) were derived from the matched point pairs via triangulation. The fundamental matrix *F* was estimated to recover both the cameras’ internal properties (e.g., focal length) and external properties (e.g., rotation and translation). Outliers and erroneous matches were filtered to improve accuracy. The third step involved estimating the 3D coordinates of corresponding points using the camera poses, resulting in a sparse point cloud. This sparse cloud was further refined through iterative optimization using Bundle Adjustment, which minimized errors across all views to ensure consistency and accuracy (Agarwal et al., 2010). The final output was generated by applying surface reconstruction methodology to 3D spatial data. The entire process yielded high-quality 3D point clouds of *C. bicolor* suitable for further analysis.

#### 2.2.3 Data preprocessing

**Figure 2:**
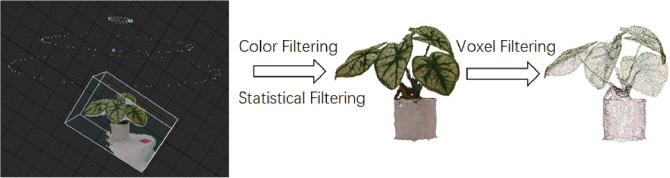
The workflow for processing point cloud data to achieve 3D reconstruction of real plant models.

We began by applying a color-threshold-based method to remove unnecessary background points, enhancing computational efficiency. Next, we applied the Statistical Outlier Removal (SOR) filter from the Point Cloud Library to remove outliers and suppress noise in the point cloud. The SOR filter assumes that the point cloud follows a Gaussian distribution, characterized by its mean *µ* and standard deviation *σ*. Outliers are identified as points with an average distance exceeding a predefined threshold. The threshold is defined by Equation 1:


(1) threshold=μ+α * σ

where *α* acts as a scaling coefficient for the standard deviation *σ*. The specific process is illustrated in **Figure 2**.

#### 2.2.4 Data manually annotation

The point cloud dataset used for network training and testing was manually annotated with semantic labels using the segmentation tool in CloudCompare. To improve the network’s generalization, mitigate overfitting, and assess segmentation performance, a total of 314 point cloud samples were manually labeled. These samples were randomly partitioned into two groups: 211 allocated for training and 103 for evaluation. Each point cloud consists of 1,024 points, with each point assigned to one of three semantic categories: leaf, stem, or non-plant.

### 2.3 Network architecture

#### 2.3.1 Backbone structure

We adopted a U-Net-structured DSN for complex prediction tasks, such as semantic segmentation, structured as a multi-scale feature integration framework incorporating cross-layer feature fusion pathways.

**Figure 3:**
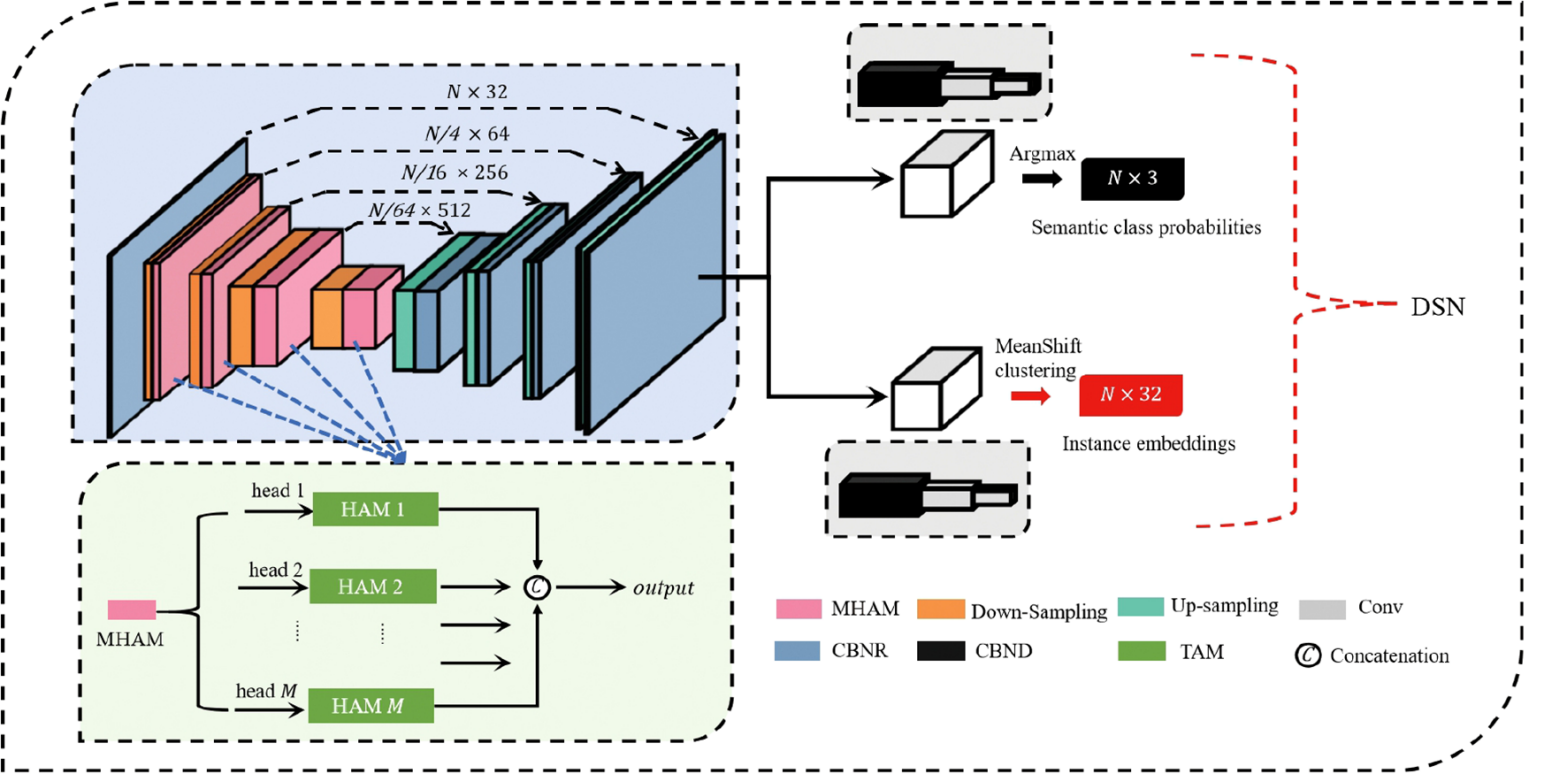
The detailed architecture of DSN for plant-part segmentation. The encoder mainly comprises four MHAM and four Down-sampling modules. The decoder mainly comprises four Up-sampling modules and four Multi-Layer Perceptron (MLP) layers. The numbers (N, D) signify the quantity of points and the output channels, respectively. FC, Fully Connected layer; DP, Dropout; MHAM, Muti-head Hierarchical Attention-based Module; Conv, convolutional layer; CBNR, Conv + BatchNorm + ReLU; CBND, Conv + BatchNorm + ReLU + DP; US, Up-sampling; DS, Down-sampling.

The proposed network is architecturally organized around three core components: the Multi-head Hierarchical Attention Module, the Down-Sampling module, and the Up-Sampling module. The detailed architecture of our DSN for point-wise segmentation is shown in **Figure 3**. Initially, the input point cloud is processed by a shared Multi-Layer Perceptron (MLP) layer for feature transformation and extraction. Subsequently, we use four encoding layers to reduce the number of point while simultaneously enriching feature complexity per point. Each encoding layer includes an MHAM and a Down-Sampling module. The point cloud undergoes four-fold downsampling, retaining just 25% of the original points at each processing layer. This results in a progressively reduced point set cardinality that decreases by factors of 4 at successive stages, ultimately reaching 1/256 of the original scale. Simultaneously, the feature dimension of each layer continuously increases to capture more information: (32 → 64 → 128 → 256 → 512). After the encoder, four decoders are used to restore the point cloud to its original number of points *N*. Each decoder layer employs an Up-Sampling module and an MLP. Through skip connections, the upsampled feature maps of the encoder’s earlier stages are fused with the decoder’s deeper stages. Finally, the DSN separates into dual pathways dedicated to semantic label prediction per point and high-dimensional instance embedding generation respectively. The final semantic predictions and instance embeddings are obtained through three shared Fully Connected (FC) layers: (*N*,128) → (*N*,32) → (*N,C*) for semantic labels, or (*N*,128) → (*N*,32) → (*N,D*) for instance embeddings. Following the initial FC layer, a stochastic masking layer is applied where half of the neural units are randomly silenced during activation. The network’s output consists of predicted semantic labels in an *N* × *C* matrix and instance embeddings in an *N* × *D* matrix, where *N* denotes the point count, with *C* and *D* corresponding to class count and embedding dimension respectively.

#### 2.3.2 Multi-head Hierarchical Attention Module

**Figure 4:**
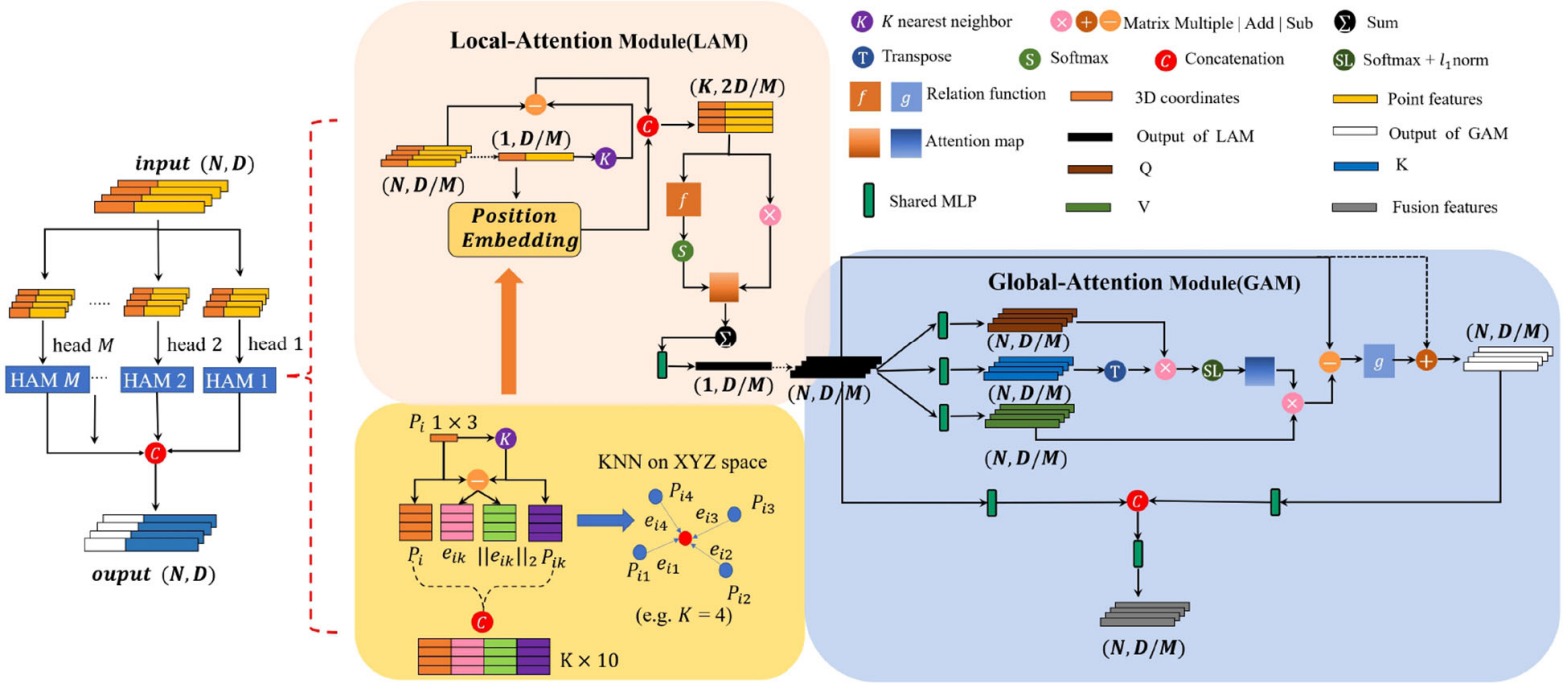
The proposed MHAM module. The top panel shows the Local Attention Module(LAM) that weights the most important features of neighboring points between local regions of ball radius, and the bottom panel shows the Global Attention Module(GAM) weights the features dependency of all points. Numbers associated with tensors denote the dimensions *N* and feature channels *D*.

We introduce a Multi-head Hierarchical Attention Module (**Figure 4**), which processes the input point cloud to capture fine-grained geometric and graph features. Each head of the MHAM consists of two sequential sub-modules: the Local Attention Module (LAM) and the Global Attention Module (GAM). Given the morphological complexity of plant point clouds, particularly scale and density variations, we employ the ball query method (Qi et al., 2017a) to identify spatially coherent neighborhoods, rather than relying on the conventional K-Nearest Neighbors (KNN) approach. The KNN is less practical for extracting features from the complex structures of plant models. The LAM focuses on the feature interdependencies within hierarchically organized local regions. These regions are determined using the ball query method, which identifies the neighboring points {*p_i_*_1_ *,p_i_*_2_ *,…,p_ik_*} (where *k* is an upper limit) of a query point *p_i_* within a metric radius *r*. On the other hand, the GAM focuses on the feature dependencies of all points, employing a self-attention mechanism to establish interconnectedness across all spatial positions of the point cloud.

#### 2.3.3 Position Embedding Module

We introduce a PEM that explicitly encodes the spatial position as shown in Equation 2:


(2) $$\text{po}{\;}_{\text{sik}}^{\;}=\text{MLP}(x_{i}⊗x_{i}^{k}⊗(x_{i}-x_{i}^{k})⊗∥x_{i}-x_{i}^{k}∥)$$

Where ${\text{pos}}_{i}^{k}$ is the positional encoding vector, *x_i_* denotes the coordinates of the central point, $x_{i}^{k}$ corresponds to those of neighboring points, and ∥·∥ computes the Euclidean distance between xi and its adjacent $x_{i}^{k}$ .

#### 2.3.4 Local Attention Module

We subsequently employed the powerful attention mechanism rather than max/mean pooling—methods prone to critical information dissipation—for automatic aggregation of the *i^th^* features $F_{i}^{k}$ . Our method constructs a local neighborhood for each center point by performing KNN search to identify its fixed *K* adjacent points. This process yields a feature set of K points, denoted as $\left\{f_{i}^{1},\ldots ,f_{i}^{K},\ldots ,f_{i}^{K}\right\}$ . Subsequently, each point’s feature fi is enhanced by concatenation with its corresponding relative feature difference ( $f_{i}-f_{i}^{k}$ ), thereby generating the augmented feature $F_{i}^{k}$ . Specifically, we employed a function *f*(·) to learn an attention score $a_{i}^{k}$ and then weighted the sum of these features, as shown in Equations 3–5:


(3) $$F_{i}^{k}=(f_{i}⊕(f_{i}-f_{i}^{k}))$$

(4) $$a_{i}^{k}=\text{softmax }(f(F_{i}^{k},W))$$

(5) $$F_{l}=\sum_{k=1}^{K}(a_{i}^{k}\cdot F_{i}^{k})$$

In this case, the function *f*(·) is implemented using a shared MLP, *W* represents the learnable weights of the MLP, and ⊕ denotes the concatenation operation.

#### 2.3.5 Global Attention Module

After aggregating the local features, we developed a Global Attention Module to refine global features through self-attention, leveraging a matrix dot-product to compute attention scores for all points. We denote the query, key, and value matrices as Q, K, and V, respectively, which are derived from the input features, as defined in Equation 6.


(6) $$Q,K,V=F_{l}\cdot (W_{q},W_{k},W_{v})$$

Here, *W_q_*, *W_k_*, and *W_v_* are the learnable weights.

To begin, we calculate the attention weights *s* by combining the Q and K matrices, and then apply the softmax operator to normalize the attention map along the first dimension, as shown in Equation 7.


(7) $$s=\text{softmax }(Q\cdot K^{T})$$

To further enhance the normalization, we apply the *l*_1_-norm to normalize the second dimension, as shown in Equation 8.


(8) $$s^{\prime }=\frac{s}{\sum_{k=1}^{K}s_{ik}}$$

The normalized attention weights $s^{\prime }$ determine the aggregated value vector output, formally denoted as *F_s_*. The difference between the self-attention feature *F_s_* and the input feature *F_l_* is quantified through element-wise subtraction. The function *g*(·) uses two shared MLPs followed by a ReLU nonlinearity. The feature fusion formula is given in Equations 9, 10:


(9) $$F_{s}=s^{\prime }\cdot V$$

(10) $$F_{i}=g(F_{l}-F_{s})+F_{l}$$

In addition, we introduced a multi-head mechanism to obtain more comprehensive information and further enhance the generalization ability of the network. This is calculated as shown in Equation 11, Where $F_{i}^{m}$ represents the feature of the $m^{th}$ head for point *p_i_*, and *M* represents the number of attention heads, which is set to 4 in this study.


(11) $$F^{\prime }=F_{i}^{1}⊕F_{i}^{m}⊕\ldots ⊕F_{i}^{M}$$

#### 2.3.6 Down-sampling module

The iterative farthest point sampling (FPS) algorithm (Qi et al., 2017b) is applied to the input point set *P*_0_ to generate the subsampled point set *P*_1_. Compared to random sampling, FPS achieves enhanced spatial coverage of point clouds when selecting equivalent numbers of centroid points, ensuring spatially uniform distribution (Qi et al., 2017b). For propagating features between the source point set *P*_0_ and the downsampled subset *P*_1_ (*P*_1_ ⊂ *P*_0_), we employ the ball query method, which establishes a fixed region scale to locate all neighboring points that form a local region for each query point in *P*_1_. The features of each local region are processed through a shared MLP, subsequently normalized via batch-wise standardization and non-linearly transformed by ReLU operations. Finally, max pooling is applied to each point in *P*_1_ using its neighboring points in *P*_0_.

#### 2.3.7 Up-sampling module

Each decoder upsampling module performs dual operations through coordinated point cloud upsampling and hierarchical feature propagation, transferring encoded representations from the subsampled set *P*_1_ to the denser superset *P*_0_ while achieving structural preservation. Similar to deconvolution in CNNs, this mechanism performs geometric upsampling of the point cloud, progressively refining abstract, holistic pattern encoding into precise, localized positional attributes. First, a multi-stage feature mapping framework is implemented, where distance-aware neighborhood interpolation operates to transfer semantic attributes between the downsampled points to the original points. Subsequently, the upsampled decoder outputs are fused with co-existing encoder states through cross-stage linkages, enabling multi-level information integration that generates enhanced descriptor volumes. The fused feature maps subsequently undergo processing through a weight-shared perceptron module, with sequential execution of batch normalization and non-linear transformation operations.

### 2.4 Multi-Value Conditional Random Field model

An MV-CRF model is constructed using the semantic labels and instance embeddings output by the DSN. Specifically, consider $P=\{p_{1},p_{2},\ldots ,p_{n}\}$ as the discrete geometric sampling of a reconstructed spatial configuration, where all spatially distributed elements constitute vertices within a complete graph topology, with all pairwise topological entities linked through bidirectional adjacency links. Each vertex has an associated semantic label *l^S^*, with $L^{S}=\{l_{1}^{S},l_{2}^{S},\ldots ,l_{n}^{S}\}$ representing the set of semantic labels. Similarly, each vertex has an instance label *l^I^*, with $L^{I}=\{l_{1}^{I},l_{2}^{I},\ldots ,l_{n}^{I}\}$ representing the set of instance labels. The graph, defined over P, L^S^, and L^I^, is referred to as MV-CRF. Combined semantic-instance segmentation is achieved by minimizing the following energy function (Equation 12), which is then solved using the mean field variational method (Blei et al., 2017).


(12) $$\begin{array}{l} E(L^{S},L^{I}❘P)=\sum_{j}\phi (l_{j}^{S})+\sum_{(j,k),j<k}\phi (l_{j}^{S},l_{k}^{S})\\ \;\;\;+\sum_{j}\text{Ψ}(l_{j}^{S})+\sum_{(j,k),j<k}\text{Ψ}(l_{j}^{S},l_{k}^{S})+\sum_{s\in S}\sum_{i\in I}\text{Φ}(s,i) \end{array}$$

Here, $\phi (l_{j}^{S})$ represents the probability of assigning point p_j_ to semantic class s; $\phi (l_{j}^{S},l_{k}^{S})$ denotes the similarity score of semantic classification between points *p_j_* and *p_k_*. $\text{Ψ}(l_{j}^{S})$ quantifies the likelihood that a vertex’s latent representation maximizes proximity to the centroid vector characterizing its associated object cohort. $\text{Ψ}(l_{j}^{S},l_{k}^{S})$ quantifies the similarity of instance labels between *p_j_* and *p_k_*, determined jointly by attributes such as position, surface normal, and color. Φ(s,i) establishes a connection between semantic and instance labels, ensuring consistency between semantic and instance predictions.

As described in the study, minimizing this energy function enforces constraints based on the semantic and physical properties of the object, thereby refining the segmentation results.

### 2.5 Loss functions

Our DSN consists of two separate branches, each responsible for a distinct task: (1) categorical classification of geometric primitives and (2) instance embedding generation at individual point resolution. The overall loss function of DSN combines the prediction loss $L_{\text{prediction}}$ and the embedding loss $L_{\text{embedding}}$ , as shown in Equation 13:


(13) $$L=L_{\text{prediction}}+L_{\text{embedding}}$$

For semantic segmentation, our implementation adopts the canonical cross-entropy formulation, formally expressed through Equation 14:


(14) $$L_{\text{sem}}=-\sum_{i=1}^{N}\sum_{j=1}^{C}p_{j}^{'}\;(i)\text{ log }p_{j}(i)$$

where *p_j_* (*i*) represents the predicted probability that the likelihood of class affiliation for the current point *i* belongs to the class *j* calculated by the model, and $p_{j}^{'}(i)$ corresponds to the reference classification indicator encoded through binary activation patterns.

Taking the instance segmentation task as an example, we used a discriminative function to represent instance embedding loss $L_{\text{embedding}}$ which is shown in Equation 15:


(15) $$L_{\text{embedding}}=\alpha \cdot L_{\text{pull}}+\beta \cdot L_{\text{push}}+\gamma \cdot L_{\text{reg}}$$

where *α*, *β*, and *γ* are hyperparameters controlling the relative weights of the pull loss ℒ_pull_, push loss ℒ_push_, and regularization loss ℒ_reg_, respectively.

The instance embedding loss consists of three components: ℒ_pull_, which pulls embeddings toward the centroids *µ_k_*; ℒ_push_, which separates the centroids from each other; and ℒ_reg_, which applies a small force to attract all centroids toward the origin. The individual components of the embedding loss are defined as follows in Equations 16–18, respectively:


(16) $$L_{\text{pull}}=\frac{1}{K}\sum_{k=1}^{K}\frac{1}{N_{k}}\sum_{j=1}^{N_{k}}{\left[∥\mu_{k}-{\text{e}}_{j}{∥}_{2}-\delta_{v}\right]}_{+}^{2}$$

(17) $$L_{\text{push}}=\frac{1}{K(K-1)}\sum_{k=1}^{K}\sum_{m=1,m\neq k}^{K}{\left[2\delta_{d}-∥\mu_{k}-\mu_{m}{∥}_{2}\right]}_{+}^{2}$$

(18) $$L_{reg}=\frac{1}{K}\sum_{k=1}^{K}∥\mu_{k}{∥}_{2}$$

where *K* specifies the instance count, ∥·∥ represents the Euclidean distance, and *N_k_* records the element count within the *k^th^* instance. The embedding of point *p_j_* is denoted by e*_j_*, while *µ_k_* and *µ_m_* represents the mean embedding of the *k^th^* instance and the *m^th^* instance, respectively. The notation [*x*]_+_ = max(0*,x*) is used to enforce non-negativity. *δ_v_* and *δ_d_* define the margin thresholds for ℒ_pull_ and ℒ_push_ respectively, with values empirically set to *δ_v_* = 0.5 and *δ_d_* = 1.5. Additionally, the weighting coefficients are fixed as *α* = *β* = 1 and *γ* = 0.001 in this study.

### 2.6 Training details

To address the challenge of feeding the entire *C. bicolor* model into point-based DL architectures—which would require a high subsampling rate and result in significant geometric information loss—we adopted the strategy used in PointNet for handling large-scale point clouds. Specifically, the input point cloud is processed using overlapping fixed-size blocks. Our proposed network processes local point clouds of 4096 points, with each point encoded as a 9-dimensional feature vector. This vector includes the centralized 3D coordinates (x, y, z), normalized color information (r, g, b), and normalized local coordinates $\left(\text{x}\prime^{\;},\text{y}\prime^{\;},\text{z}\prime^{\;}\right)$ . The neural network independently segments the plant parts for each local point cloud during model training. For testing, an unseen dataset is preprocessed in the same manner, and the final segmentation predictions from all blocks are merged to achieve complete segmentation result.

### 2.7 Experiments

#### 2.7.1 Experiments setup

The neural network was developed within the PyTorch framework, employing a Stochastic Gradient Descent (SGD) optimizer configured with momentum = 0.9 and weight_decay_ = 0.0005 during training. The learning rate commences at 10^−3^, undergoing a halving process every 20 epochs. The network was trained with a batch size of 16 across 100 epochs. The experiments were conducted using PyTorch 1.6 on a 64-bit Linux CentOS 8 server equipped with an AMD EPYC 7302 CPU (16 cores, 3.00 GHz), 256 GB of RAM, and two NVIDIA GeForce RTX 3090 GPUs. The dataset used for the experiments was the *C. bicolor* point cloud dataset collected and annotated as described earlier.

#### 2.7.2 Evaluation metrics

This study conducts a comprehensive assessment of the proposed method’s efficacy across both point-wise and object-wise dimensions.

For evaluating semantic segmentation, we employed widely used metrics, including overall accuracy (oAcc), mean of Intersection over Union (mIoU), and mean of class-wise accuracy (mAcc) across all classes. These metrics are commonly utilized for assessing 3D point cloud segmentation performance. The formulas for the OAcc, mAcc, and mIoU are as follows in Equations 19–21, respectively:


(19) $$\text{oAcc}=\frac{\sum_{i=0}^{c}p_{ii}}{\sum_{i=0}^{c}\sum_{j=0}^{c}p_{ij}}$$

(20) $$\text{mAcc}=\frac{1}{c+1}\sum_{i=0}^{c}\frac{p_{ii}}{\sum_{j=0}^{c}p_{ij}}$$

(21) $$\text{mIoU}=\frac{1}{c+1}\sum_{i=0}^{c}\frac{p_{ii}}{\sum_{j=0}^{c}p_{ij}+\sum_{j=0}^{c}(p_{ji}-p_{ii})}$$

where *c* is the category of structural parts of plants, therefore we set *c* = 3 in this study. Here, *p_ij_* represents class-*i* ground truth instances misclassified as class *j*, while *p_ji_* corresponds to class-*j* instances erroneously predicted as class *i*; both terms quantify cross-classification errors. The elements *p_ii_* indicate correctly classified instances within their true categories.

For instance segmentation, the evaluation was based on the mean precision (mPrec), mean recall (mRec) with an IoU higher than 0.5 or 0.25 in each semantic category (Conn et al., 2017). Furthermore, we treat instance segmentation as a form of object detection and employ average precision (AP) with an IoU threshold of 0.5 (Pham et al., 2019) as the evaluation metric. The formulas for the Prec, Rec, mPrec, and mPrec are as follows in Equations 22–25, respectively:


(22) $$\text{Prec}=\frac{❘TP_{c}^{ins}❘}{❘PR_{c}^{ins}❘}$$

(23) $$\text{Rec}=\frac{❘TP_{c}^{ins}❘}{❘GT_{c}^{ins}❘}$$

(24) $$\text{mPrec}=\frac{1}{❘c❘}\sum_{i=1}^{c}\frac{❘TP_{i}^{ins}❘}{❘PR_{i}^{ins}❘}$$

(25) $$\text{mRec}=\frac{1}{❘c❘}\sum_{i=1}^{c}\frac{❘TP_{i}^{ins}❘}{❘GT_{i}^{ins}❘}$$

Here, $❘TP_{c}^{ins}❘$ represents the count of successfully predicted instances with an IoU greater than 0.5 relative to the ground truth and belonging to semantic class *c*. $❘PR_{c}^{ins}❘$ and $❘GT_{c}^{ins}❘$ denote the number of predicted instances and ground truth instances, respectively, for semantic class *c*. The term |*c*| indicates the number of semantic classes, which in this study is ❘c❘=3 . The ground truth instances are categorized into semantic classes *c* ∈ {non-plant, leaf, stem}. The Precision-Recall (P-R) curve is obtained by plotting Precision (Prec) on the vertical axis against Recall (Rec) on the horizontal axis. The Average Precision (AP) is defined as the area under the P-R curve and is computed as follows Equation 26):


(26) $$AP=\int_{0}^{1}Prec(Rec)\;d\text{R}e\text{c}$$

#### 2.7.3 Semantic segmentation experiments

We performed a thorough quantitative and qualitative evaluation of our per-point semantic segmentation method, benchmarking it against several mainstream deep learning models: (1) PointNet (Qi et al., 2017a), (2) PointNet++ (Qi et al., 2017b), (3) DGCNN (Wang et al., 2019b), (4) ShellNet (Zhang et al., 2019), (5) PointWeb (Zhao et al., 2019), (6) PointTransformer (PCT) (Zhao et al., 2021), (7) JSNet (Zhao and Tao, 2020), (8) ASIS (Wang et al., 2019a), and (9) JSIS3D (Pham et al., 2019). Among these, models (1)–(6) support only semantic segmentation and were trained and tested using semantic labels alone. In contrast, our proposed network and models (7)–(9) — designed for joint semantic and instance segmentation — were evaluated under unified annotation conditions, where identical semantic and instance labels were utilized throughout both training and testing procedures.

#### 2.7.4 Instance segmentation experiments

We performed comprehensive qualitative and quantitative evaluations against state-of-the-art multi-task models, assessing instance segmentation performance using mPrec, mRec, and AP as key metrics. These metrics were calculated with IoU thresholds of 0.5 and 0.25 for each semantic category. An instance point group is considered a valid segmentation region if its IoU exceeds the predefined threshold. For instance segmentation comparison, we evaluated ASIS (Wang et al., 2019a), JSIS3D (Pham et al., 2019), and JSNet (Zhao and Tao, 2020) on the test set of the point cloud data. Additionally, we evaluated two variations of our pipeline: “MHA-CRF” which incorporates MV-CRF, and “MHA-MSC” which applies the Mean-Shift Clustering (MSC) algorithm (Comaniciu and Meer, 2002) directly to DSN’s instance embeddings.

#### 2.7.5 Ablation experiments

Ablation experiments were conducted to assess the impact of PEM, LAM, and GAM modules within the DSN framework on semantic and instance segmentation performance.

For the Neighbors Number, we investigated the optimal number of neighboring points *k*, which defines the local region range for feature extraction based on the attention mechanism.

For the Position Embedding Module, we conducted a thorough investigation into the impact of different spatial information representations on our framework, particularly focusing on ablative experiments for the PEM. These experiments were divided into the following cases:

P1: Encodes only the 3D coordinates of the point *x_i_*.

P2: Encodes only the 3D coordinates of the neighboring points *x_k_*.

P3: Encodes the 3D coordinates of the point *x_i_* and its neighboring points *x_k_* as [*x_i_* ⊕ *x_k_*].

P4: Encodes the 3D coordinates of the point *x_i_*, its neighbors *x_k_*, and their relative positions (*x_i_*− *x_k_*) as [*x_i_* ⊕ *x_k_* ⊕ (*x_i_* − *x_k_*)].

P5: Encodes the 3D coordinates of the point *x_i_*, its neighbors *x_k_*, and the Euclidean distance $∥x_{i}-x_{k}∥$ as $\left[x_{i}⊕x_{k}(x_{i}-x_{k})\right]$ .

P6: Encodes the 3D coordinates of the point *x_i_*, its neighbors *x_k_*, the relative positions (*x_i_*− *x_k_*), and Euclidean distance $∥x_{i}-x_{k}∥$ as $\left[x_{i}⊕x_{k}⊕(x_{i}-x_{k})⊕∥x_{i}-x_{k}∥\right]$ .

## 3 Results

### 3.1 Semantic segmentation performance

The quantitative performance of semantic segmentation, evaluated using metrics (oAcc, mAcc, and mIoU) on the test set, is presented in **Table 1**. Each row in the table represents the experimental results for the corresponding model. Our proposed model demonstrates leading segmentation results, attaining 99.16% oAcc, 95.73% mAcc, and 93.64% mIoU, while showing enhanced capabilities in local information perception and segmentation accuracy.

**Table 1:** Quantitative results of different DL-based models on our labeled plant cloud point dataset.

Methods	Acc(%)			IoU(%)			oAcc(%)	mAcc(%)	mIoU(%)
	Leaf	Stem	Non-plant	Leaf	Stem	Non-plant			
PointNet (Qi et al., 2017a)	91.71	72.49	98.12	88.76	67.75	86.51	91.38	87.44	81.01
PointNet++ (Qi et al., 2017b)	98.45	84.44	99.94	95.74	80.40	99.57	96.76	94.28	91.90
DGCNN (Wang et al., 2019b)	97.80	85.09	97.91	94.03	77.79	95.06	95.43	93.60	88.96
ShellNet (Zhang et al., 2019)	98.66	79.96	99.97	97.68	66.33	98.99	98.19	92.86	87.67
PointWeb (Zhao et al., 2019)	98.48	82.43	99.74	94.61	79.77	99.43	93.52	93.55	91.27
PCT (Zhao et al., 2021)	99.63	83.70	99.69	97.88	72.18	99.65	98.41	94.34	89.90
ASIS (Wang et al., 2019a)	97.68	82.88	**99.98**	96.85	68.06	98.98	97.34	93.51	87.96
JSNet (Zhao and Tao, 2020)	97.47	86.80	98.75	96.37	65.67	97.34	97.07	94.35	86.46
JSIS3D (Pham et al., 2019)	98.99	74.64	99.76	98.17	69.91	98.96	98.56	91.13	89.01
DSN (Ours)	**99.63**	**87.58**	99.97	**98.86**	**82.19**	**99.89**	**99.16**	**95.73**	**93.64**

The best-performing values are highlighted in bold.

The improved performance arises from the integration of the LAM and GAM modules within the network, which systematically combines neighborhood and global information. These enhancements address common challenges in 3D point cloud segmentation, particularly in handling boundary regions and areas with sparse plant biomass.

As shown in **Table 1**, the accuracy for the stem class consistently falls behind that of the other two classes across all models in the semantic segmentation task. We identify two main factors contributing to this phenomenon. First, the stem constitutes a relatively inconspicuous plant structure, with its points representing a limited percentage of the whole. This limited representation reduces the number of true positive predictions for stem points. Consequently, each false negative prediction significantly impacts the stem’s segmentation accuracy. Second, the stem’s diverse and complex structure poses additional challenges for accurate segmentation.

**Figure 5:**
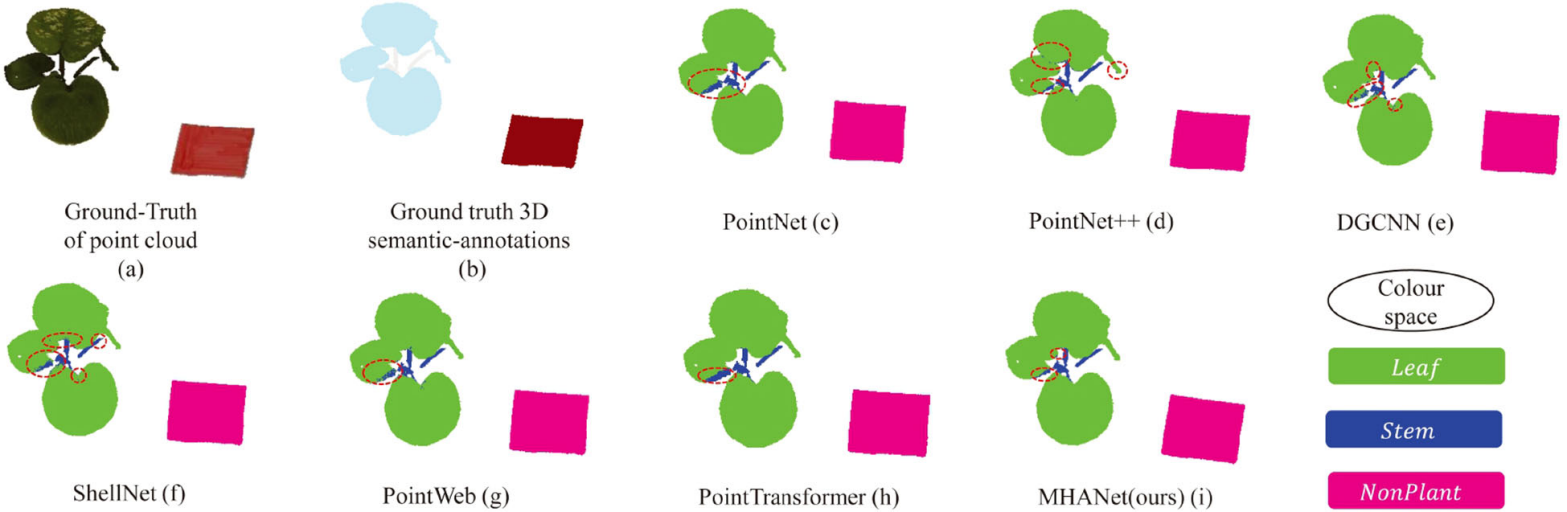
Visualization comparison of semantic segmentation results between mainstream deep learning models and the proposed DSN on the *C. bicolor* point cloud dataset. **(a, b)** represent the ground truth: the raw point cloud and the point cloud with semantic labels, respectively. **(c–h)** show the semantic segmentation outcomes for each model, where different colors distinguish categories, and misclassified points are highlighted with the red circles.

Additionally, **Figure 5** presents a visual comparison of semantic segmentation outcomes across various DL-based networks on a sample point cloud, highlighting the strengths of our approach.

### 3.2 Instance segmentation performance

**Figure 6:**
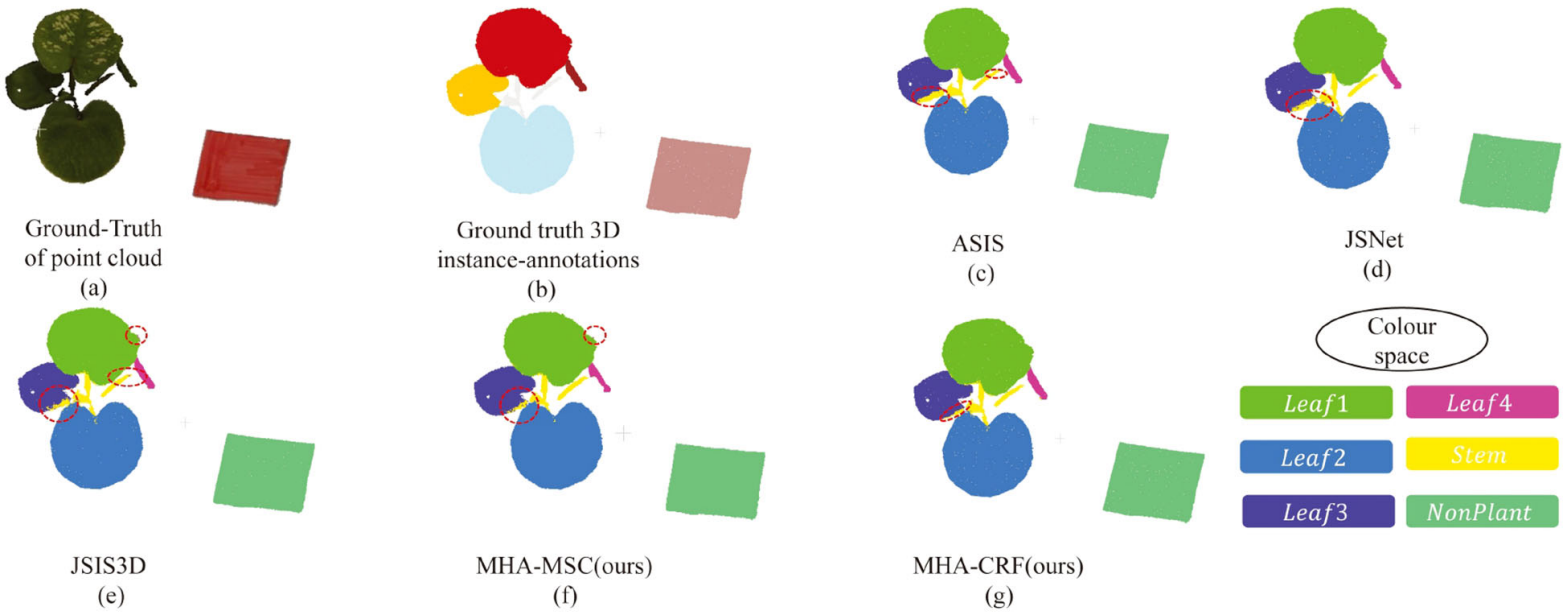
Visualization comparison of instance segmentation results between mainstream deep learning models and the proposed DSN on the *C. bicolor* point cloud dataset. **(a, b)** represent the ground truth: the raw point cloud and the point cloud with instance labels, respectively. **(c–g)** show the instance segmentation outcomes for each model, where different colors distinguish categories, and misclassified points are highlighted with the red circles.

As evidenced in **Table 2**, our proposed method attains superior instance segmentation performance across all categories relative to existing deep learning models. Notably, MHA-CRF significantly improves segmentation performance in certain categories over DSN alone. **Figure 6** provides a visual comparison of instance segmentation results from our proposed DSN network and other mainstream DL models on the *C. bicolor* point cloud dataset, highlighting the enhanced accuracy and robustness of our approach.

**Table 2:** Performance of instance segmentation on our labeled point cloud dataset.

Methods	IoU_0.5_ (%)			IoU_0.25_ (%)		
	mPrec	mRec	mAP	mPrec	mRec	mAP
ASIS (Wang et al., 2019a)	73.19	62.43	54.69	86.51	71.73	66.49
JSNet (Zhao and Tao, 2020)	77.06	67.25	55.40	85.75	74.85	68.51
JSIS3D (Pham et al., 2019)	76.11	63.92	52.90	87.72	72.80	68.02
MHA-MSC (ours)	84.17	**72.66**	66.16	93.34	**79.59**	78.03
MHA-CRF (ours)	**87.94**	72.36	**71.61**	**95.68**	77.20	**79.21**

We also present the standalone performance of DSN using the MSC algorithm (denoted as MHA-MSC) and its results when running the full pipeline with CRF (denoted as MHA-CRF).

The best-performing values are highlighted in bold.

### 3.3 Ablation study

#### 3.3.1 Ablated modules of network

As shown in **Tables 3**, **4**, the full DSN (A1) consistently achieved the best performance, with a semantic segmentation mIoU of 93.64% and instance segmentation mAP of 71.61%. Removing GAM (A2) led to the most significant drop in global feature quality, reducing leaf IoU by 5.64% and mAP by 12.35%. Disabling LAM (A3) particularly affected instance segmentation, lowering mAP to 60.78%. Without PEM (A4), accuracy and mIoU also declined, confirming its role in maintaining structural integrity. These results highlight the importance of all three modules in achieving optimal segmentation performance.

**Table 3:** Performance of instance segmentation on our labeled point cloud dataset.

Method	Architecture			Acc(%)			IoU(%)		
	PEM	LAM	GAM	leaf	stem	non-plant	leaf	stem	non-plant
A1	✓	✓	✓	**99.63**	**87.58**	**99.97**	**98.86**	**82.19**	**99.89**
A2	✓	✓	X	95.93	84.35	99.83	93.22	72.94	98.79
A3	✓	X	✓	97.06	80.39	99.95	94.13	74.14	98.91
A4	X	✓	✓	94.03	80.31	99.98	91.10	80.63	99.63

The symbol ✓ indicates the inclusion of a module, while X denotes its removal. The best-performing values are highlighted in bold.

**Table 4:** Ablation study on oAcc and mIoU for semantic segmentation in the proposed DSN.

Method	Architecture			Acc(%)			IoU(%)		
	PEM	LAM	GAM	leaf	stem	non-plant	leaf	stem	non-plant
A1	✓	✓	✓	**99.16**	**95.73**	**93.64**	**93.64**	**89.00**	**90.50**
A2	✓	✓	X	94.85	93.37	88.32	88.32	85.00	86.50
A3	✓	X	✓	95.55	92.47	89.06	89.06	84.50	87.00
A4	X	✓	✓	93.24	91.44	90.45	90.45	86.00	88.50

The symbol ✓ indicates the inclusion of a module, while X denotes its removal. The best-performing values are highlighted in bold.

#### 3.3.2 Neighbors number

As presented in **Table 5**, setting *k* to 16 yielded the best semantic and instance segmentation performance across most categories. The findings from our experiments suggest that with insufficient values of the neighbor count *k*, the model struggles to effectively acquire adequate local patterns and contextual relationships necessary for precise prediction outcomes. Conversely, when *k* is too large, each attention layer tends to introduce excessive noise from potentially less relevant points, increasing computational costs and reducing model accuracy.

**Table 5:** The effect of setting different k on network segmentation performance.

Segmentation task type	Metrics		Different *k* values				
			*k*=4	*k*=8	*k*=16	*k*=32	*k*=64
Semantic Segmentation	Acc(%)	leaf	97.04	97.91	**99.63**	98.67	98.05
		stem	77.57	83.31	**87.58**	85.29	80.68
		non-plant	**99.98**	99.96	99.97	99.96	**99.98**
	IoU(%)	leaf	93.97	95.13	**98.86**	95.25	94.41
		stem	72.58	81.76	**82.19**	80.68	76.20
		non-plant	99.53	98.94	**99.89**	98.32	96.75
	oAcc(%)		95.42	96.29	**99.16**	96.36	95.72
	mAcc(%)		91.53	93.73	**95.73**	94.64	92.90
	mIoU(%)		88.69	91.94	**93.64**	91.42	89.12
Instance Segmentation	mPrec(%)		82.24	83.23	**87.94**	84.40	85.64
	mRec(%)		72.13	73.73	72.36	**73.76**	70.47
	*mAP*_0.5_(%)		67.20	65.53	**71.61**	69.08	67.26
	mPrec(%)		89.29	91.11	**95.68**	90.16	92.96
	*m*Rec(%)		77.31	80.17	77.20	**78.57**	75.76
	*mAP*_0.25_(%)		74.91	76.38	**79.21**	78.79	77.84

Bold values indicate optimal performance metrics.

#### 3.3.3 Position embedding

**Table 6** compares the effect of each PEM configuration on semantic segmentation performance in our network using the *C. bicolor* point cloud dataset. The results show that explicitly encoding all spatial information (P6) yields the best segmentation performance for both leaf and stem classes across all metrics. The inclusion of relative position (*x_i_*− *x_k_*) is particularly impactful, as it enables the network to better capture local geometric patterns.

**Table 6:** The ablation analysis of Acc, IoU, oAcc, mAcc, and mIoU for the effect of the PEM on the semantic segmentation performance.

PEM	Acc (%)			IoU (%)			oAcc (%)	mAcc (%)	mIoU (%)
	Leaf	Stem	Non-plant	Leaf	Stem	Non-plant			
P1	98.45	70.59	99.89	94.70	69.02	99.80	95.99	89.64	87.74
P2	95.66	79.93	99.97	92.75	72.78	99.66	94.51	91.85	88.40
P3	97.94	77.89	99.95	94.75	74.29	99.81	96.01	91.93	89.61
P4	98.72	82.80	**99.98**	95.66	80.51	99.54	96.71	93.83	91.90
P5	98.52	80.21	99.94	95.01	78.34	99.65	96.20	92.89	91.00
P6	**99.63**	**87.58**	99.97	**98.86**	**82.19**	**99.89**	**99.16**	**95.73**	**93.65**

The best-performing values are highlighted in bold.

## 4 Discussion

This study attempts to utilize a hardware platform designed for plant cultivation experiments to generate 3D reconstructed point cloud datasets from 2D image data of real plant models. We then applied a newly proposed DSN to achieve semantic segmentation of various plant organs. Through this automated, non-destructive, and high-throughput pipeline, we successfully captured key phenotypic traits of real plants. This approach demonstrates significant practical value in applications such as seeding phenotype measurement. To enable this, we first developed an image capture platform capable of obtaining multi-view 2D image sequences of real plants in a controlled growth environment. Using 180 images captured from three angles, we reconstructed a 3D point cloud dataset for real plant models. After data preprocessing and manual annotation, we curated a complete dataset for open research, consisting of 276 point cloud samples.

Inspired by the attention mechanism, we proposed DSN to achieve high-precision semantic and instance segmentation on labeled point clouds. Our DSN demonstrated superior performance on the *C. bicolor* point cloud dataset, achieving an oAcc of 99.16%, mAcc of 95.73%, and mIoU of 93.64%, surpassing mainstream point-based deep learning models such as PointNet. For instance segmentation, which we treated as an object detection task, we evaluated performance using AP at IoU thresholds of 0.5 and 0.25. We further improved instance segmentation performance using MV-CRF, by predicting class labels and embedding points into high-dimensional vectors Compared with other existing deep learning models, including ASIS, our DSN achieved the best instance segmentation results, with mPrec, mRec, and mAP reaching 87.94%, 72.36%, and 71.61%, respectively, at an IoU threshold of 0.5 on the *C. bicolor* dataset.

For instance segmentation tasks, PointNet lacks a dedicated module for local feature extraction (Qi et al., 2017b), which limits its ability to capture the geometric characteristics of stems and leaves, resulting in a relatively low mIoU of around 80%. PointNet++ achieves the second-best results, with oAcc/mAcc/mIoU values of 96.76%, 94.28%, and 91.90%, respectively. PointNet++ leverages a hierarchical feature extraction process, organizing local regions based on a metric radius (Qi et al., 2017b). This flexible design allows for versatile adjustments outside the network’s framework, a strategy that is also incorporated into our proposed network. PointWeb ranks next, effectively combining global shape and local neighborhood features (Zhao et al., 2019). Compared to PointNet, it improves mAcc and mIoU by approximately 5% and 10%, respectively. ShellNet and DGCNN, which use K-neighborhoods instead of metric radii for hierarchical point grouping and feature aggregation, offer less flexibility in adjusting receptive field dimensions. This limitation can hinder performance when accounting for both plant structure and data density. Bifunctional networks like ASIS tend to perform relatively poorly on semantic tasks, as they must balance semantic and instance segmentation tasks during training. In contrast, our proposed DSN maintains an effective balance between these tasks while achieving superior semantic segmentation results.

The attention mechanism employed in this network results in moderately higher computational demands compared to traditional convolution and MLP-based approaches (Zhao et al., 2021). For input data sized (1, 1024, 9), PointNet requires 2.4G FLOPs with 3.55M parameters, while PCT operates at 4.38G FLOPs with 2.93M parameters yet delivers highly accurate results. Our MHANet utilizes 20.84G FLOPs with 5.71M parameters. Although demanding the highest computational and memory resources among all models, MHANet achieves superior performance - improving stem accuracy by 4 percentage points and IoU by 10 percentage points over PCT.

Comprehensive experimental evaluations reveal that integrating attention mechanisms leads to noticeable improvements in plant-part segmentation accuracy, effectively mitigating the prevalent under-segmentation challenges in 3D point cloud processing.

Notably, most point cloud segmentation techniques require large volumes of fully labeled data Chen et al. (2023); Zhang et al. (2023). To reduce the labor-intensive nature of data annotation, future research could explore weakly supervised or unsupervised learning methods for plant-part segmentation. To further enhance the self-adaptability of fully automated phenotypic measurements, addressing the cross-cutting nature of plant components will be a key focus moving forward.

The results presented in this paper establish the foundational conditions necessary for achieving fully automated intelligent phenotypic measurements. Real-time automated detection of plant phenotypic traits facilitates the analysis of plant growth status and enables the derivation of digital growth patterns (e.g., leaf color variation over time, timing and location of new leaf emergence, and senescence patterns of older leaves). Based on these growth patterns, we can establish evaluation metrics (such as leaf coloration and total leaf area) and configure varying resource environments. Through controlled experiments, we thereby identify optimal growth conditions that consistently regulate the expression of plant traits via environmental controls. Furthermore, deviations from established growth patterns may indicate nutrient deficiencies, disease outbreaks, or pest infestations. For ornamental plants, quantifying such growth patterns helps determine peak ornamental periods. This informs commercial sales timing strategies, mitigating losses from missed optimal selling windows. Additionally, advancements in drone and 3D technologies now enable the acquisition of 3D point clouds for structurally complex plants in field environments. We plan to adapt this methodology to staple crops in future work, facilitating enhanced yields and improved pest and disease control.

## Data Availability

The original contributions presented in the study are included in the article/**Supplementary Material**. Further inquiries can be directed to the corresponding author.
