# Supplementary figures and images for: A dual-task segmentation network based on multi-head hierarchical attention for 3D plant point cloud
Source: Front Plant Sci. 2025 Jul 22;16:1610443. doi: 10.3389/fpls.2025.1610443 (PMC12321799; doi:10.3389/fpls.2025.1610443)

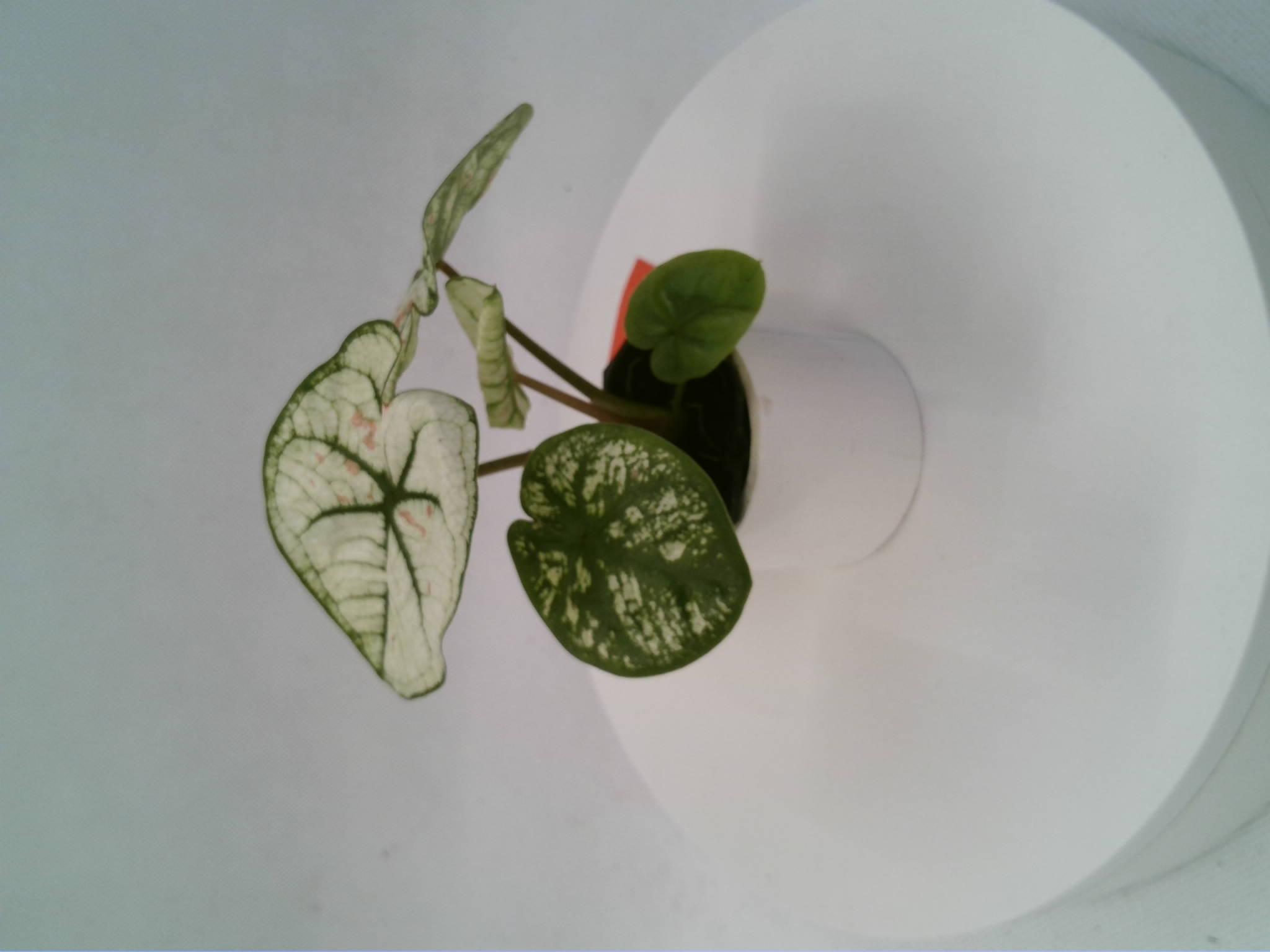

Supplement: Supplementary file 1 [file Image1.jpeg]

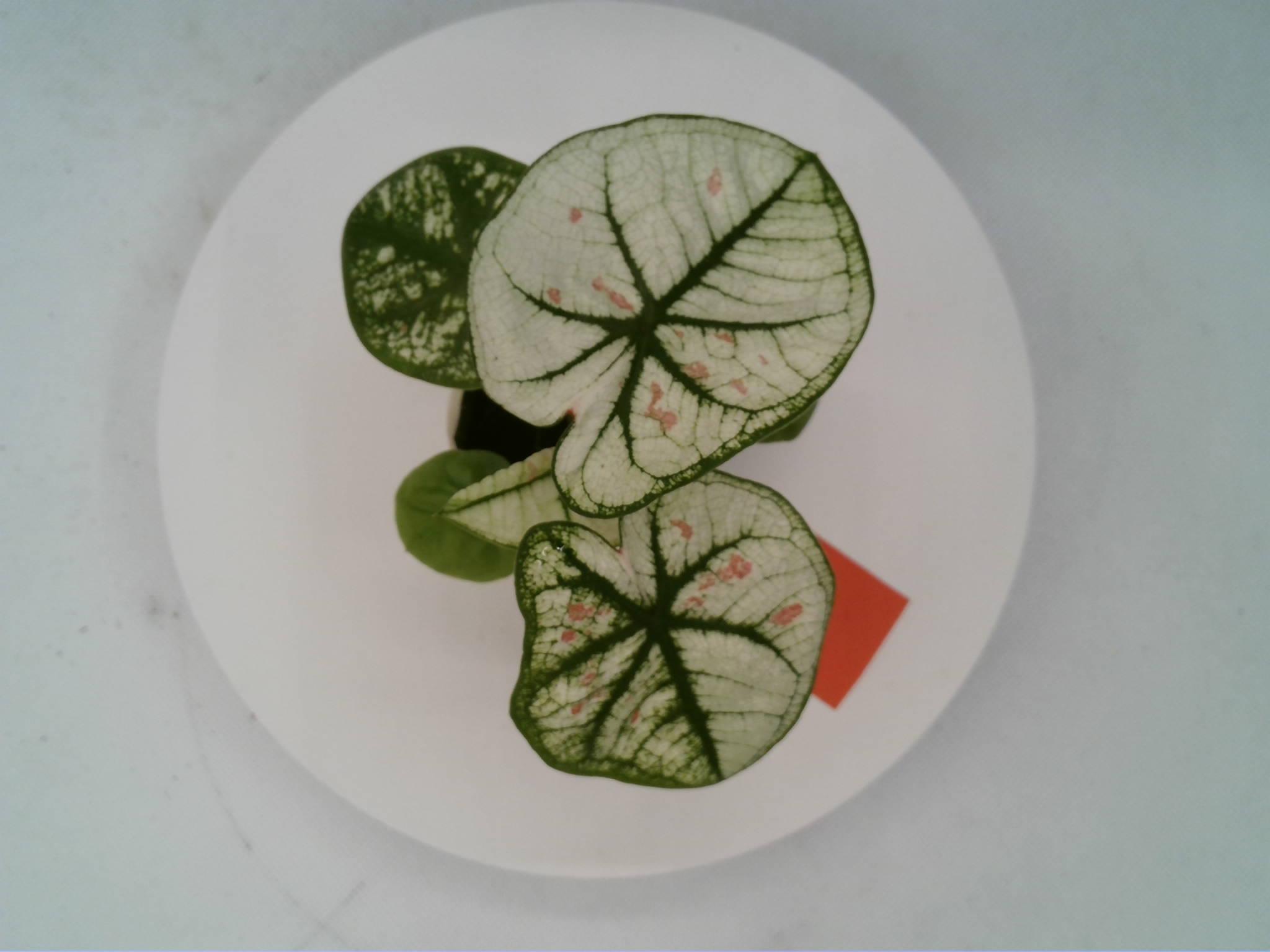

Supplement: Supplementary file 2 [file Image2.jpeg]

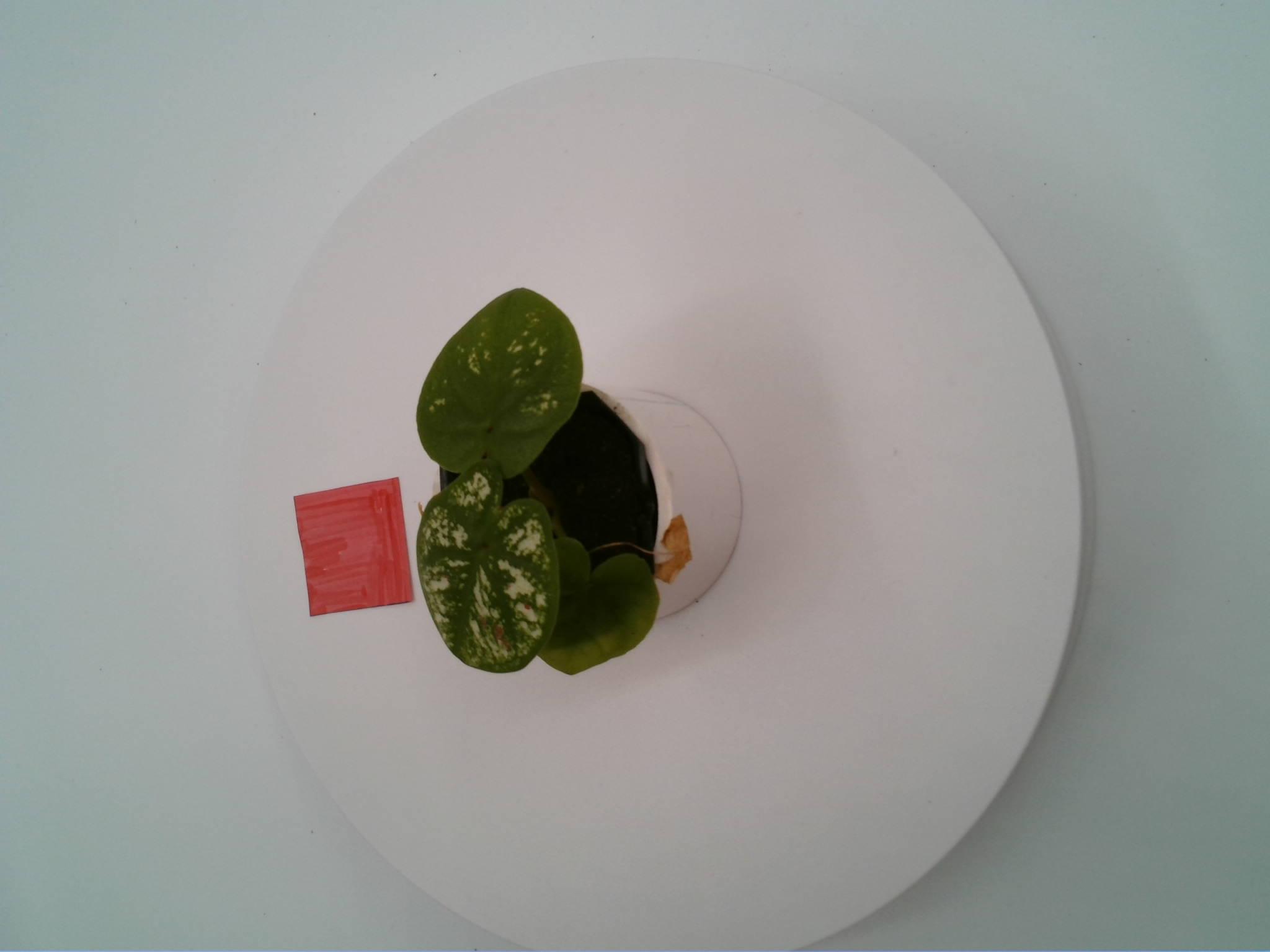

Supplement: Supplementary file 3 [file Image3.jpeg]

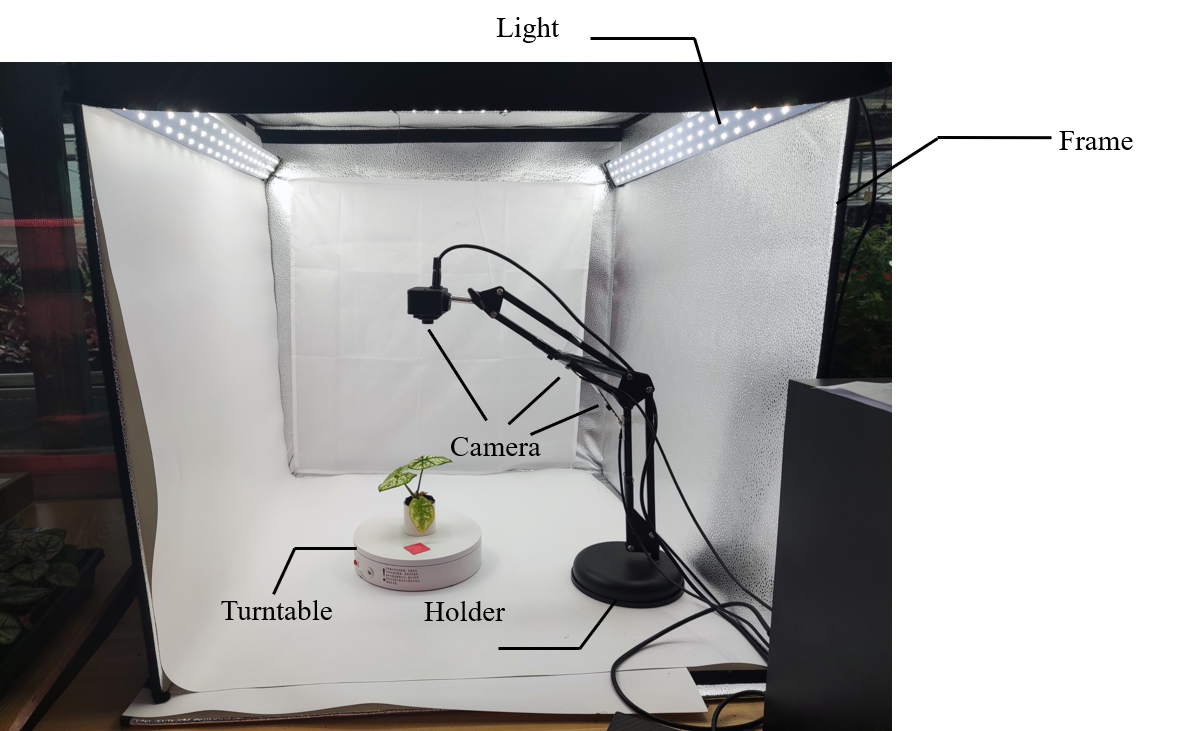

Supplement: Supplementary file 4 [file Image4.png]
